# Supplementary material for: GMM-Based Expanded Feature Space as a Way to Extract Useful Information for Rare Cell Subtypes Identification in Single-Cell Mass Cytometry
Source: Int J Mol Sci. 2023 Sep 13;24(18):14033. doi: 10.3390/ijms241814033 (PMC10531342; doi:10.3390/ijms241814033)
Supplement: Supplementary file 1 [file ijms-24-14033-s001.zip › ijms-2553922-supplementary.pdf]

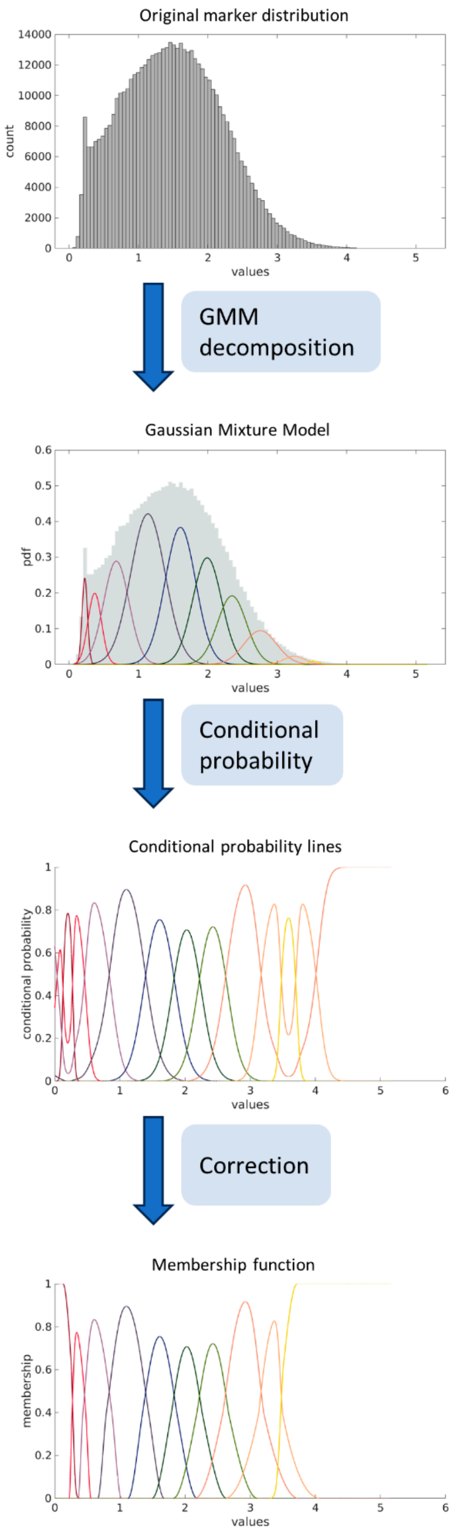

Supplementary Figure S1. The data distribution for each marker is subjected to Gaussian Mixture Model (GMM) decomposition, yielding a set of components that potentially represent distinct cell groups characterized by specific expression values. Conditional probabilities are computed and visually depicted in the form of lines. In the next step, the lines are corrected, leading to the membership function that determines the assignment of each cell to each of the components (new features).

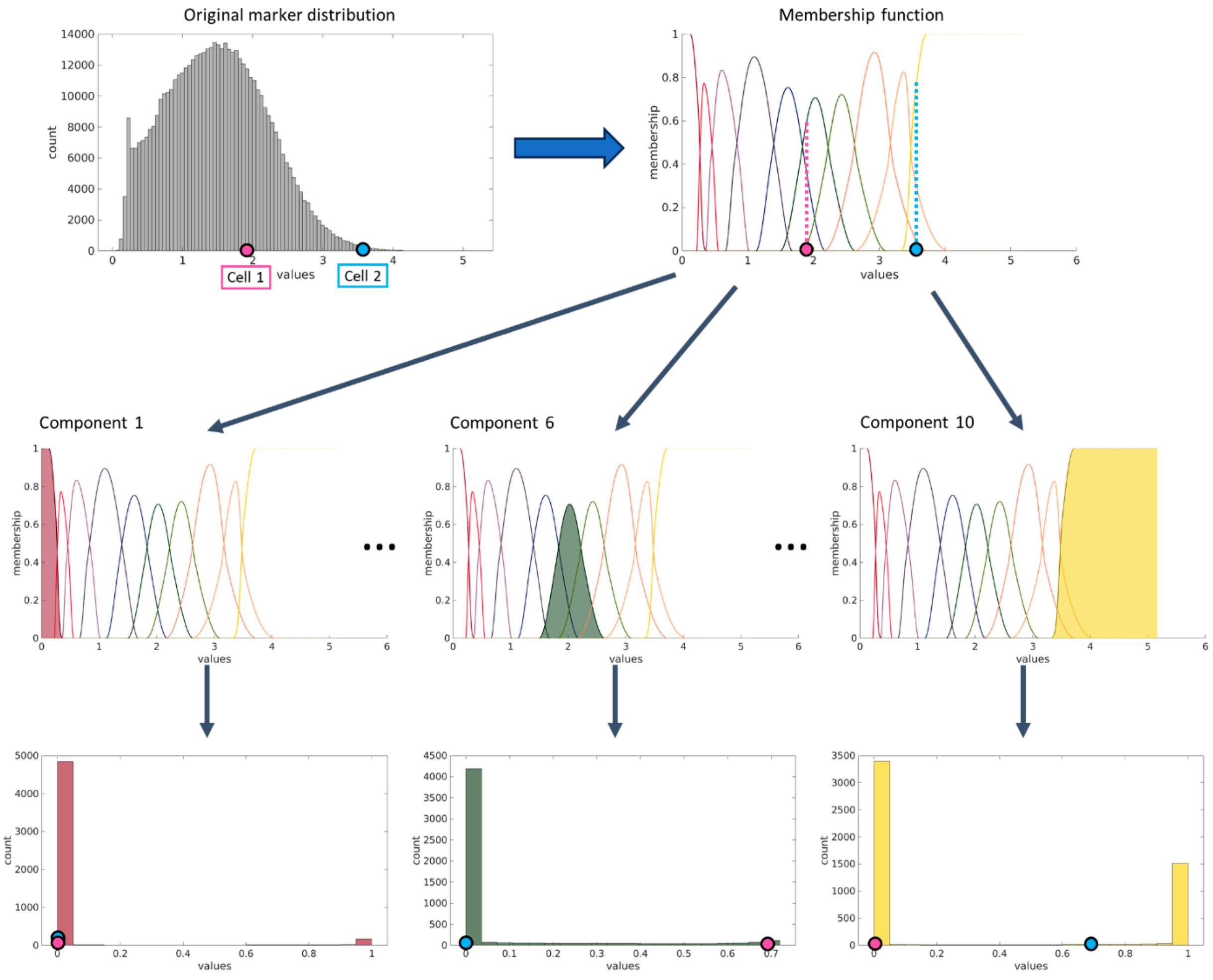

Component	1	2	3	4	5	6	7	8	9	10
Cell 1	0	0	0	0	0.20	0.69	0.11	0	0	0
Cell 2	0	0	0	0	0	0	0	0.03	0.25	0.70

Supplementary Figure S2. The idea behind the expanded feature space. For each marker, the membership function is created. Each component is a new feature that represents the assignment of each cell to the component. The new features have a significantly different distribution than the original marker. The new feature values are in the range [0,1], where 0 means no expression of the feature and 1 - an assignment only to one component (great distance of cell to other components). The blue and pink dots represent different cell types characterized by various components, therefore, are easier to distinguish in the expanded feature space.

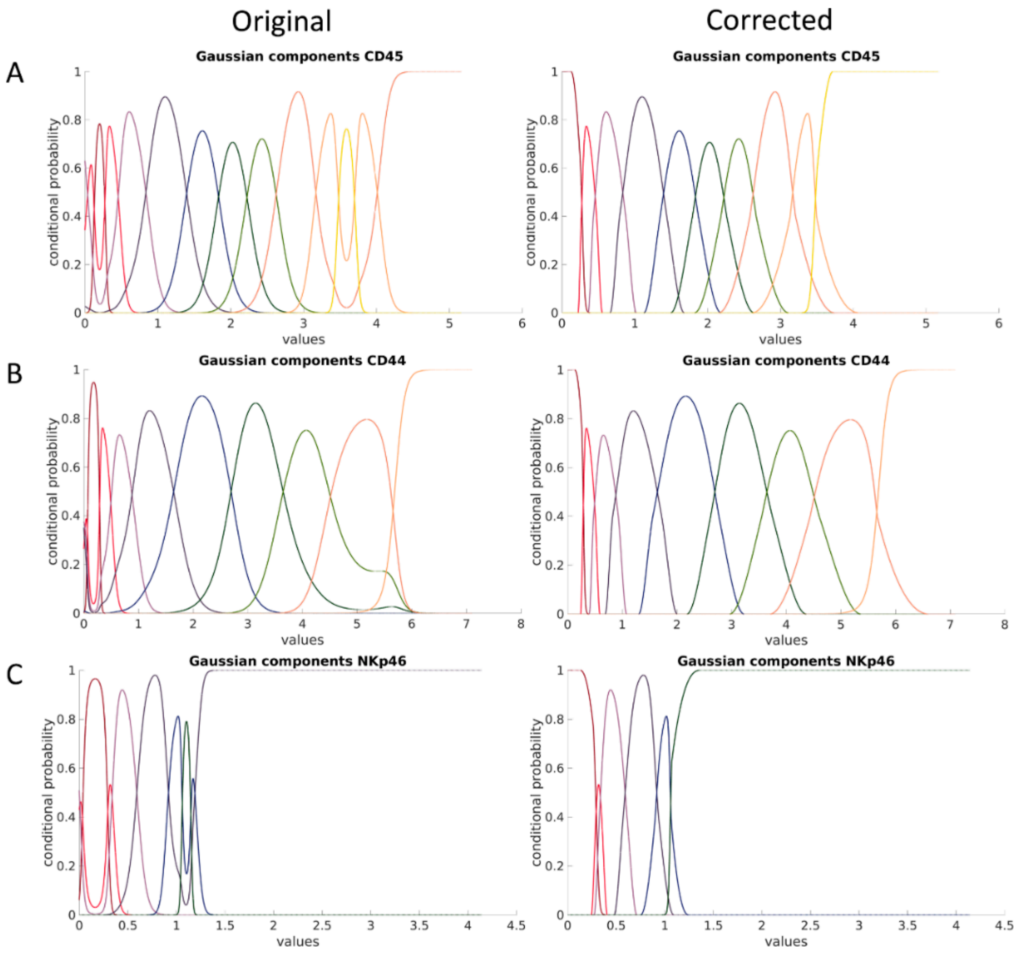

Supplementary Figure S3. The effect of the correction algorithm to transform conditional probabilities from Gaussian Mixture Models into membership function.
